# Animal-assisted therapy on happiness and life quality of chronic psychiatric patients living in psychiatric residential care homes: a randomized controlled study

**DOI:** 10.1186/s12888-020-02980-8

**Published:** 2020-12-01

**Authors:** Mohammad Sahebalzamani, Omid Rezaei, Ladan Fattah Moghadam

**Affiliations:** 1 Department of Management, Faculty of Health, Tehran Medical Sciences, Islamic Azad University, Tehran, Iran; 2 Department of Psychiatry, University of Social Welfare and Rehabilitation Sciences, Tehran, Iran; 3 Department of Nursing, Faculty of nursing and midwifery, Tehran Medical Sciences, Islamic Azad University, Tehran, Iran

**Keywords:** Psychiatric patients, Animal-assisted therapy, Happiness, Convalescent hospitals, Quality of life

## Abstract

**Background:**

Psychiatric patients who live in psychiatric residential care homes may often feel a loss of autonomy, decision making, and participation in social activities. They usually have few or no visitors and also do not have any purpose for living. Animals may increase the happiness and quality of life of psychiatric patients. This study aimed to evaluate the effects of Animal-Assisted Therapy (AAT) on happiness and quality of life of chronic psychiatric patients living in psychiatric residential care homes in Tehran, Iran.

**Methods:**

This randomized controlled trial was conducted with 70 males with a chronic psychiatric disorder who were living in psychiatric residential care homes in Tehran, Iran, in 2016. The patients were randomly selected and divided into animal therapy intervention group and control group. Patients in the intervention group received animal-therapy with a bird for eight weeks. Patients in the control group received no intervention. The Oxford Happiness Inventory evaluated all patients pre and post-intervention. To evaluate life quality, the Wisconsin Quality of Life Index was used. Data were statically analyzed using SPSS Ver.19.0. ANCOVA with pretest statistical control. The significance level was set as *p* < 0.05.

**Results:**

The mean age in both control and intervention groups were 47.12 and 45.82 years, and the mean age of illness onset for both control and intervention groups was 18.94 and 16.83 years, respectively. The result of this study showed that happiness in the intervention group had significantly increased (*p* < 0.001). The results also showed that the quality of life in four sub-domains increased significantly.

**Conclusion:**

To bring happiness to chronic psychiatric patients living in psychiatric residential care homes is essential and may result in returning them to society and healthy life. The results of this study showed that AAT was helpful for chronic psychiatric patients living in psychiatric residential care homes and not only made them happy but also increased their quality of life.

**Trial registration:**

This was registered in Iranian Registry of Clinical Trials (IRCT) (clinical trial code: IRCT20101013004922N4. Registered 2018-08-19. Retrospectively registered, https://www.irct.ir/trial/32390

## Background

The level of happiness and continuing a normal life among psychiatric patients is important for both patients and their families, which have been neglected over recent years and can be an important criterion for recovery and treatment in these patients [1, 2]. Happiness is an essential need for human beings and has a significant effect on the formation of personality and mental health [3]. According to Cohen, happiness is a positive concept that is significant for health and vital to preserve it [4]. Happiness is defined as a fundamental emotion characterized as a permanent state which is combined with: the absence of negative emotions, the presence of positive emotions, life satisfaction, social engagement, and objectives in life [5].

Skinner et al. believed that quality of life is a sense of well-being in life [6]. The World Health Organization (WHO) declared that the quality of life has many domains, including; physical health, psychological health, level of independence, social relations, environmental factors, and personal beliefs, and it can be assessed by different means [7]. Quality of life is a broad concept and covers all aspects of life [8]. A general interpretation of the concept is that quality of life is dictated by individual personal characteristics, conditions in life, and a subjective view of the current situation and must be defined by the patient’s frame of reference [9]. The quality of life for people with psychiatric disorders is worse than for the general population, and human life expectancy is shorter [10].

The human-to-human relationship is highly significant to create a positive emotional relationship and live happily; however, psychiatric patients are not able to communicate appropriately [11, 12]. The relationship between humans and animals can be useful for their health as much as a human-to-human relationship [13].

Animal Assisted Therapy (AAT) is a purpose-oriented and designed therapeutic intervention controlled and provided by health, education, and human service professionals. AAT is provided by a formally educated (with active licensure) expert with experience within the scope of the professionals’ practice which considers improving the physical, cognitive, behavioral, and/or socio-emotional functioning of the participants [14].

The relationship between animals and patients provides an environment that causes better communication of the patients, enhances their self-confidence, reduces illness symptoms, and leads to an increase in happiness in life [15, 16]. AAT is a method that can resolve depression and loneliness caused as a result of living in residential care centers, and enhances happiness and satisfaction with life [17]. In this therapeutic method, animals like dogs, horses, cats, and birds are usually used [15].

In a study by Calvo et al., they concluded that the AAT program was a useful adjunct to conventional psychosocial rehabilitation in patients with schizophrenia. They also reported a remarkable decrease in cortisol levels after the intervention [18].

Taziki et al. showed that AAT enhanced and had a positive effect on cognitive, social, communicative, and behavioral functions among children with autism spectrum disorder^15^. In a study by Nurenberg et al., the effect of AAT on psychiatrically hospitalized patients was evaluated; authors concluded that AAT was an effective therapeutic modality for chronic psychiatric patients [19]. Another study by Vrbanac et al., which evaluated the effects of the presence of animals on loneliness, showed a significantly lower score in the UCLA Loneliness Scale [20].

Regarding the increasing number of psychiatric patients, increased hospitalization costs, separation from the family, long period of illness, and the importance of happiness in life, it is necessary to replace a lower-cost method such as AAT in order to bring happiness and pleasure to psychiatric the lives of psychiatric patients living in residential care centers [21]. Therefore, the aim of the present study was to evaluate the effects of AAT on happiness and quality of life of chronic psychiatric patients living in psychiatric residential care homes in Tehran, Iran.

## Methods

### Setting and study design

This randomized controlled trial was performed in the Welfare of Shahriar city and its affiliated chronic psychiatric care centers. The study time was from late February in 2016 to August of 2016. Both two non-profit facilities were located in Shahriar city. The residents of the facilities had the same length of stay, level of education, and pet ownership. The veteran’s facility had a higher number of males and the males were older than the other facility. Since these centers were both governmental had the same practitioners, the same number of staff and also the patients were identical in diagnosis.

### Sample size and study sampling

Two centers from eight 24-h care centers for chronic psychiatric patients were selected by single-stage cluster random sampling. The first psychiatric residential care home was assigned as the intervention center, and the second as the control one. The patients who met the inclusion criteria were enrolled. The written consent form was obtained and signed by their guardians.

To determine the required sample size at 95% confidence level and power of the test of 0.8%, it is assumed that the magnitude of the effect of AAT on the quality of life of chronic psychiatric patients residing in the care center in the intervention group, in comparison to the control group, has at least the score of d = 0.7, in the following formula:

$$ \mathrm{n}=\frac{{\left({Z}_{1-\frac{\alpha }{2}}+{Z}_{1-\beta}\right)}^2}{d^2}+1 $$

**Fig. 1:**
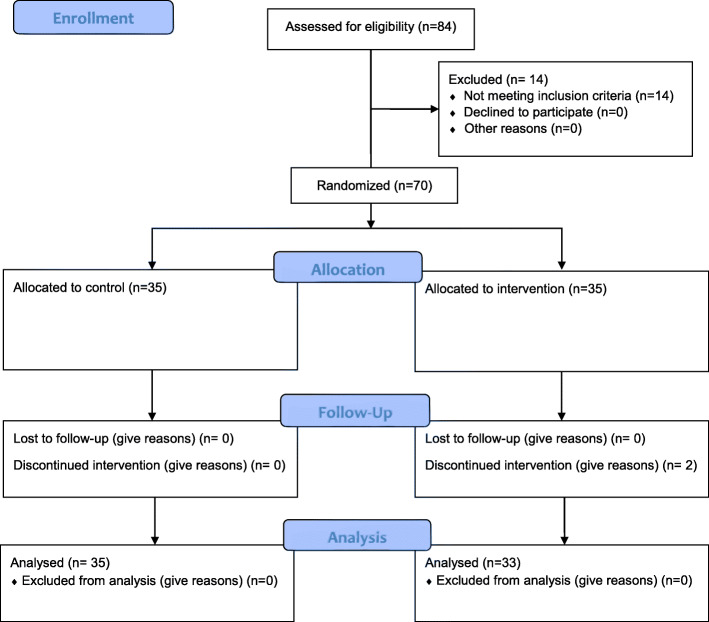
Flow diagram of the progress through the phases of two-group parallel randomized trial

The sample size was calculated as 33 in the intervention group and 33 in the control group. It should be noted that based on similar studies, the standard deviation of quality of life was estimated to be 14.5 and with the probability of a sample dropout of 10% it was added to the above sample size. Therefore, the sample size in each group was determined to be *n* = 35 (Fig. 1).

$$ \boldsymbol{n}=\frac{\mathbf{2}{\left(\mathbf{1.96}+\mathbf{0.84}\right)}^{\mathbf{2}}}{{\mathbf{0.7}}^{\mathbf{2}}}=\mathbf{32}+\mathbf{1} $$

### Inclusion and exclusion criteria

The following characteristics were considered to select the research samples:

1. Diagnosis of chronic mental illness by a specialized team of psychiatrists

2. The minimum residence of 6 months or more in the same psychiatric care center

3. Having the ages 18–60 years

4. Not having mental retardation

5. Able to complete the questionnaire

6. Willingness to participate in the study and to keep animals (budgerigar)

7. Having the ability to participate in research (ability to perform animal care activities such as giving water and food and cleaning birdcages)

8. Having no allergies and not being afraid of the animal

9. The patient and their caregivers’ satisfaction to participate in the research

### Study instruments

We used two questionnaires in our study. The Oxford Happiness Inventory (OHI) was used as a data collection tool. According to the earlier discussion by Argyle and Crossland (1987), happiness is constructed by three main areas: the frequency and intensity of positive affect or joy, the average level of satisfaction over a period, and the lack of negative feelings including depression and anxiety. They measured these three components using a 29-item questionnaire and the respondents are asked to select one of four options which is scored using 4-point scale (always, sometimes, seldom, and never) [22].

Scores > 22 show low happiness, scores from 22 to 44 is average happiness, scores 44 to 68 show high happiness, and scores 68 to 87 show very high happiness [23, 24]. The reliability of this instrument was calculated as 90% using Cronbach’s Alpha [25]. To investigate the quality of life, Wisconsin quality of life index (W-QLI) was used. It is a multidimensional model for measuring patients’ quality of life and consists of eight domains, including; satisfaction with life domains, occupational activities, symptoms, physical health, social relations, finances, psychological well-being, and activities of daily living [26]. Information on important aspects of life are classified into eight sections:

1. Satisfaction level: Includes 9 questions about patient satisfaction with life, how to spend time, loneliness, housing, neighborhood condition, type of food and clothing, medical and psychiatric services, transportation access and marital life.

2. Occupational Activities: Includes 4 questions that deal with the patient’s main activities (work, education, housework), and the patient’s feelings about the main activities and their extent.

3. Mental Health and relaxation: Includes 11 questions that ask about the patient’s feelings over the past 4 weeks regarding emotional and psychological states such as happiness, loneliness, boredom, restlessness, feelings of pride, confusion, excitement, and depression.

4. Symptoms: It consists of 5 questions about the patient’s condition and feelings about the symptoms of his illness. Getting high scores in this area is the sign of controlling the symptoms of the illness.

5. Physical health: There are 2 questions. One question is about the patient’s perception of his physical condition and the other question measures the extent of one’s feelings about physical health.

6. Social Relationships: Includes 7 questions that measure patient satisfaction with the number of friends, how to deal with them, relationships with family, and coping with them.

7. Financial Status (money): This section has 4 questions that determine the financial adequacy and patient satisfaction with the amount of money available.

8. Activities of daily living: It consists of 8 questions and examines the activities of daily living during the past 4 weeks and measures the extent of participation and support of others and the existing problems while performing activities.

### Validity and reliability of the instrument

Scientific validity is the true and correct measurement of the variables studied in such a way that just the considered item is measured, not anything else. The generalizability of the study results to the larger community is also termed validity (Hejazi, 2014, p. 95). The validity of this questionnaire has been examined and verified by Hojati Abedi [27] through content validity. In this study, the internal consistency of Wisconsin Quality of Life Questionnaire index was obtained through Cronbach’s alpha for each section as follows: life satisfaction level = 93%, occupational activity level = no reliability (because patients in these centers lacked occupation), symptoms = 82.5%, mental health = 63%, physical health = unreliable, drugs = unreliable, social support = 77.8%, financial status = 73%, daily living activities = 69%.

Oxford Happiness Inventory was completed by both intervention and control groups before the intervention, and the scores for both groups were recorded [25].

### Procedure

Ethics approval was granted by the University Ethics Committee of Faculty of Nursing and Midwifery of Islamic Azad University, Tehran Medical Sciences Branch. After selecting two centers, the researcher referred to these centers for data collection and obtained permission from the related authorities. The patients who met the inclusion criteria were selected and after obtaining their consent and getting written consent from their legal caregivers, the study commenced. Completion of the questionnaire required 30 to 60 min depending on the patient’s ability and condition. The questionnaire was filled out by the patient himself with the presence and supervision of the researcher and in most cases by the researcher through the interview which took 2 months. In order to avoid spreading the information, patients in one center (Behnoud center) were selected as intervention groups (35 patients) and patients in other center (Saman center) were selected as the control group (*n* = 35) randomly. The required arrangements were made for the intervention group to initiate the program of keeping a pet animal. The AAT program was administered to the intervention group by the researcher who had already received the requisite training on how to care for and interact with the pet (budgerigar) by a veterinarian. Before starting the program for the intervention group, required explanations were provided on how to participate in keeping the animal and taking care of it, and necessary recommendations were offered to all individuals in the intervention group by the researchers in order to observe hygiene and prevent possible diseases, that they must wash their hands thoroughly before and after interacting with pets and clean their hands with an alcohol-based solution if they have been in direct contact with the pet. It should be noted that the considered animals were mature and were examined by a veterinarian to ensure the health of the animal before being given to the patients, and the relevant licenses issued by the veterinarian were provided to the authorities of the center and all the required tests and examinations were performed on them for participating in AAT program. After 8 weeks, Wisconsin quality of life questionnaire was again completed for both intervention and control groups and their quality of life was determined. The intervention and control groups were compared before and after the intervention. Pre-test and post-test were used to determine the effect of animal care on improving the quality of life of these patients and to test the research assumptions.

### Intervention

After obtaining the consent of each participant in the intervention group at Behnood Care Center, the program was administered by giving each patient a budgerigar (two patients were given a pair of budgerigars in a cage) and they took care of the budgerigars for an eight-week period (in the presence of the director of the center and the researcher) at defined office hours (7 am to 1 pm) and did the pet care-related activities such as giving them food and water, cleaning the birdcage and so on. Patients eagerly went to the care center in the morning to take care of their birds and brought the cages in the 5 h, and they sat beside the cages for a long time and talked to each other and to the researcher about the birds, the memories and experiences they had about keeping the birds in the past. Most patients, who were often lying on their beds and had nothing to do, got out of their beds to watch the pets and take care of their affairs. At the end of the shift, the birds were taken to the determined room in the care center by the patients themselves (in the absence of the researcher, patients could visit their birds in the presence of the care center staff). In the meantime, the researcher also monitored the accuracy of the tasks and the way patients took care of the birds. Throughout the whole eight-week intervention period, meetings with the same budgerigars were held to establish appropriate interactions and communications. All participants were also assured that this was voluntary and that they could leave the study if they did not wish to continue.

### Animal welfare

The researchers made sure that during the intervention all the rights of animals be reserved and no harms are inflicted on the birds.

### Statistical analysis

We used SPSS Ver.19.0 for the data analysis, applying descriptive and inferential methods. We carried out an independent samples t-test to compare the level of happiness and quality of life dimensions for the control and intervention groups. Since only two measurements were conducted, differences in scores in happiness level from baseline through post-measurement were assessed between the two groups by using analysis of covariance (ANCOVA) with baseline values of happiness level, respectively, as covariates. The significance level was set as *p* < 0.05. The results of Levene’s test on the homogeneity of error variances were not significant (*p* ≥ 0.05). In the most cases to carry out the statistical analysis concerning the mean of two or several societies, the distribution of test statistics is determined by assuming that their variance is the same. Therefore, before running the mean tests, the equality of variances in societies should be examined by the Levene’s Test. The Kolmogorov-Smirnov test showed the normality of data distribution (*p* ≥ 0.05).

## Results

The results showed that the mean age in both control and intervention groups was 47.12 and 45.82 years, and the mean age of illness onset for both control and intervention groups was 18.94 and 16.83 years, respectively. (Table 1)

**Table 1:** Descriptive statistics of demographic data of two groups

Variables	Control Group Mean (SD)	Intervention Group Mean (SD)
Age (Year)	47.12 (10.68)	45.82 (8.95)
Age of illness onset (Year)	18.94 (11.58)	16.83 (5.27)
Hospitalization duration (Months)	22.31 (15.57)	21.10 (11.47)
Number of Hospitalization	4.37 (3.25)	4.51 (4.82)

The result showed that happiness in the intervention group was significantly increased in post-test scores (*p* < 0.001). (Table 2)

**Table 2:** Mean and standard deviation of happiness level among patients in the intervention and the control groups in pre- and post-tests

Stage	Group	Happiness level Mean (SD)	*P-*Value
Pretest	Intervention	77.05 (19.23)	
	Control	96.33 (13.96)	
*P-*Value	0.12		
Post-test	Intervention	87.88 (17.61)	0.001
	Control	65.26 (15.65)	
*P-*Value	0.001		

After the pretest, statistical control between the intervention and the control groups were significantly different in the post-test (*p* ≤ 0.05). (Table 3)

**Table 3:** The results of ANCOVA for quality of life and happiness level

Source	SS	df	MS	F	P	Partial Eta square	Observed power
Pretest	7043.62	1	7043.22	39.84	0.001	0.373	0.996
Group	499.95	1	4119.95	23.03	0.001	0.258	0.996
Error	11,845.65	67	176.81	–	–	–	–

The results showed that the quality of life of the patients before and after the intervention in the control group was not significantly different in any of the domains (*p*-value < 0.05). The quality of life of the patients before and after the intervention in the intervention group showed that in the satisfaction with life domains (*p* = 0.009), psychological well-being (*p* = .0012), and activities of daily living (*p* = 0.003) there was a significant difference. (Table 4)

**Table 4:** The results of dependent t-test for comparing the quality of life of patients in both intervention and control groups before and after the intervention

Quality of Life Domains	Time	Intervention Group			Control Group		
		Mean (SD)	t	p	Mean (SD)	t	p
satisfaction with life domains	Before	49.78 ± 16.82	−3.24	.009*	48.68 ± 16.25	−1.06	.323
	After	51.51 ± 16.93			49.67 ± 16.34		
occupational activities	Before	47.10 ± 11.58	−.945	.213	46.15 ± 11.54	−.984	.414
	After	47.13 ± 11.65			46.14 ± 11.50		
psychological wellbeing	Before	64.89 ± 12.87	−2.741	.012*	63.89 ± 12.87	1.202	.274
	After	66.15 ± 13.08			63.91 ± 12.90		
symptoms	Before	54.57 ± 16.65	−1.041	.102	54.59 ± 16.65	.741	.512
	After	54.08 ± 16.52			54.58 ± 16.65		
physical health	Before	54.75 ± 14.31	−1.122	.098	53.65 ± 14.31	.874	.312
	After	54.25 ± 14.41			53.66 ± 14.39		
social relations	Before	60.39 ± 14.21	.451	.184	60.35 ± 14.21	1.025	.198
	After	60.59 ± 14.26			60.37 ± 14.25		
finances	Before	67.21 ± 23.38	−.741	.741	66.28 ± 23.38	−1.121	.127
					66.21 ± 23.29		
	After	67.13 ± 23.4					
activities of daily living	Before	47.10 ± 11.58	−3.295	.003*	46.85 ± 11.58	1.954	.214
	After	48.53 ± 11.88			46.95 ± 11.67		

## Discussion

In the present study, we evaluated the effect of Animal Assisted Therapy on the level of happiness and the quality of life of patients with chronic psychiatric disorders living in psychiatric residential care homes. In our study, the mean age in both control and intervention groups was 47.12 and 45.82 years, respectively. In a randomized controlled trial study by Calvo et al., which was conducted on patients to evaluate the effect of AAT as a useful psychological rehabilitation, the mean age of patients was 47.8 years [18]^,^ which was almost the same as ours.

The results of the present study showed that AAT was effective in psychiatric patients who mostly had schizophrenia, and their happiness level was significantly increased. Calvo et al. also found that AAT was effective in patients with schizophrenia [18].

Animal-assisted therapeutic programs and keeping pets are increasingly used in different communities, cities, nursing homes, rehabilitation centers, and hospitals [28]. Using animals in therapy sessions leads to a remarkable drop in stress, anxiety, depression, fear, and loneliness among patients [29, 30].

In a study by Paula Calvo et al., animal-assisted therapy reduced the symptoms of schizophrenia and stress in the patients [18]. In our study, we showed that the budgerigars brought about happiness in the living environment of the psychiatric patients who mostly had schizophrenia. It seems that the presence of an animal can affect an individual’s loneliness and make him/her happy.

Happiness depends on the individual’s personal attitude and perception and refers to a pleasant state that is caused by positive emotions [5]. AAT is among those modalities which decrease loneliness and brought about happiness, although the exact mechanism by which AAT decreased loneliness is unclear [31]. One hypothesis is that the human becomes attached to the AAT animals [32]. The attachment has been defined as an emotional bond that supports a sense of closeness, well-being, and security [33]. In our study, patients live with birds for 8 weeks and seem that bird keeping leads to an emotional bond between psychiatric patients and birds. Consequently, they feel better and make them happy.

An unexpected observation to be mentioned was by the low rate of brief conversation during AAT because we assumed that there might be a lot of quick “chatting” as residents were handed animals, gave them back. The other unanticipated observation was also the high rate of long conversation during AAT —the staff, volunteers, and residents spent much more time talking when an animal was involved. According to previous studies [34], the role of the animals was the facilitators for human reciprocal action. It may be because staff and residents remembered pets, or were passionate to dedicate time to spend with the animals, so they engaged in longer conversations. This finding illustrates that AAT provided residents extended, and more effective conversations and the quality of interaction may be improved during AAT. In addition, the lower rate of conversation during AAT may demonstrate that participants were engaged in longer conversations, or required for brief exchanges. Our findings suggest that in future studies of the health advantages of AAT, terms such as “social interaction” should be carefully defined to have more impacts.

The required period for prove the potential benefits of human–animal interaction may be minimal [35]. Previous studies suggested that mental health inpatients with mood and psychotic disorders showed significant decline in anxiety after a 30-min AAT session [36, 37]. Another research has reported that interaction with a dog reduced anxiety in hospitalized patients with physical illness [38]. According to these studies, recent results proved that AAT improves the educational sector through use of sessions with AAT as a stress and anxiety reduction approach [39].

We found that the quality of life of the patients before and after the intervention in the intervention group in the satisfaction with life domains, psychological well-being, and activities of daily living there was a significant difference, which indicated the intervention effects on the quality of life of the patients.

Therefore, we conducted the present study to investigate the impact of AA, and the outcome treatment, on the understanding of satisfaction and on the physical and psychic wellbeing the individuals have experienced through the scale through observation. Our results revealed that animal-assisted therapy applied a positive influence on recipients. Our findings are in line with previous studies using other animals, including horses [16], dolphins [40] and cats [41], which reported beneficial effects on physical, behavioral, and psychological symptoms, resulting in an improved quality of life.

The usefulness of AAT may be a result of individual’s positive and emotional responses to an animal [42]. When AAT is used in chronic psychiatric patients care the human–animal bond is used to diminish symptoms and increase social involvement and communication, which is believed may improve the QoL of patients

A study by Berget et al. concluded that animal-assisted intervention with farm animals might reduce depression and state anxiety and increase self-efficacy in patients with psychiatric disorders [43]. In another study by Berget et al., a significant increase in self-efficacy in the treatment group was reported [10].

Pedersen et al., in a clinical trial, showed a significant decline in depression and a significant increase in self-efficacy in patients with depression [44].

It seems that animal therapy is effective in patients with psychiatric disorders.

### Study limitation

The present study had a few limitations which should be acknowledged. One of the main limitations of our study was randomization in one center and each group. Therefore, this randomization may have distorted the effects of the intervention. Another limitation was that the mean pre-intervention happiness score in the control group was much higher than the intervention group and that the score went down, therefore we controlled this effect by using the ANCOVA analysis. Additionally, we ran the t-test analysis on the same material without using Bonferroni correction, so this can be a bias.

## Conclusion

Considering ways to enhance happiness in chronic psychiatric patients living in nursing homes is important and have a significant impact on the improvement of their quality of life. The results of this study showed that AAT was effective in psychiatric patients, and not only their happiness scale increased but also their quality of life increased.

## Data Availability

The datasets used and/or analyzed during the current study are available from the corresponding author on reasonable request.

## Abbreviations

QOL: Quality of Life
AAT: Animal-Assisted Therapy
WHO: World Health Organization
OHI: Oxford Happiness Inventory
W-QLI: Wisconsin quality of life index

## References

[1] Boyer L, Millier A, Perthame E, Aballea S, Auquier P, Toumi M (2013). Quality of life is predictive of relapse in schizophrenia. BMC Psychiatry.

[2] Noroozi M, Alibeigi N, Armoon B, Rezaei O, Sayadnasiri M, Nejati S, Fadaei F, Ghahestany DA, Dieji B, Ahounbar E (2018). Patterns of relapse risks and related factors among patients with schizophrenia in razi hospital, Iran: a latent class analysis. Pol Psychol Bull.

[3] Veenhoven R (2008). Healthy happiness: effects of happiness on physical health and the consequences for preventive health care. J Happiness Stud.

[4] Cohen S (2002). Happiness and the immune system. Positive Health.

[5] Cloninger CR, Zohar AH (2011). Personality and the perception of health and happiness. J Affect Disord.

[6] Skinner EA, Steinwachs DM, Handley K, Lehman A, Fahey M, Lyles CA (1999). Met and unmet needs for assistance and quality of life for people with severe and persistent mental disorders. Ment Health Serv Res.

[7] Organization WH (2014). Development of a global mental health action plan 2013–2020.

[8] Oort FJ, Visser MR, Sprangers MA (2005). An application of structural equation modeling to detect response shifts and true change in quality of life data from cancer patients undergoing invasive surgery. Qual Life Res.

[9] Coppock V, Dunn B (2009). Understanding social work practice in mental health: sage.

[10] Berget B, Ekeberg Ø, Braastad BO (2008). Animal-assisted therapy with farm animals for persons with psychiatric disorders: effects on self-efficacy, coping ability and quality of life, a randomized controlled trial. Clin Pract Epidemiol Ment Health.

[11] Berry K, Barrowclough C, Haddock G (2010). The role of expressed emotion in relationships between psychiatric staff and people with a diagnosis of psychosis: a review of the literature. Schizophr Bull.

[12] Rezaei O, Bayani A, Mokhayeri Y, Waye K, Sadat Y, Haroni J, Latifi M, Mohammadi R, Ghaffari M, Noroozi M (2018). Applying psychoeducational program on general health and communication skills in caregivers of patients with schizophrenia: a randomized controlled trial. Eur J Psychiatry.

[13] Birke L, Bryld M, Lykke N (2004). Animal performances: an exploration of intersections between feminist science studies and studies of human/animal relationships. Fem Theory.

[14] Jegatheesan B: IAHAIO WHITE PAPER.

[15] Evans N, Gray C (2012). The practice and ethics of animal-assisted therapy with children and young people: is it enough that we don't eat our co-workers?. Br J Soc Work.

[16] Dimitrijević I (2009). Animal-assisted therapy–a new trend in the treatment of children and addults. Psychiatr Danub.

[17] Nazarian Z, Armoon B, Rezaei O, Banihashem S, Hamideh M (2018). The effect of pet therapy concurrent with common medication on positive, negative, cognitive and motor symptoms of schizophrenia: a randomized control trial. Pol Psychol Bull.

[18] Calvo P, Fortuny JR, Guzmán S, Macías C, Bowen J, García ML, Orejas O, Molins F, Tvarijonaviciute A, Cerón JJ (2016). Animal assisted therapy (AAT) program as a useful adjunct to conventional psychosocial rehabilitation for patients with schizophrenia: results of a small-scale randomized controlled trial. Front Psychol.

[19] Nurenberg JR, Schleifer SJ, Shaffer TM, Yellin M, Desai PJ, Amin R, Bouchard A, Montalvo C (2014). Animal-assisted therapy with chronic psychiatric inpatients: equine-assisted psychotherapy and aggressive behavior. Psychiatr Serv.

[20] Vrbanac Z, Zečević I, Ljubić M, Belić M, Stanin D, Brkljača Bottegaro N, Jurkić G, Škrlin B, Bedrica L, Žubčić D (2013). Animal assisted therapy and perception of loneliness in geriatric nursing home residents. Coll Antropol.

[21] Steel Z, Marnane C, Iranpour C, Chey T, Jackson JW, Patel V, Silove D (2014). The global prevalence of common mental disorders: a systematic review and meta-analysis 1980–2013. Int J Epidemiol.

[22] Francis LJ, Katz YJ (2000). Internal consistency reliability and validity of the Hebrew translation of the Oxford happiness inventory. Psychol Rep.

[23] Hills P, Argyle M (2002). The Oxford happiness questionnaire: a compact scale for the measurement of psychological well-being. Personal Individ Differ.

[24] Sabet M, LOTFI KF (2010). Standardization of Oxford happiness inventory.

[25] Liaghatdar MJ, Jafari E, Abedi MR, Samiee F (2008). Reliability and validity of the Oxford happiness inventory among university students in Iran. Span J Psychol.

[26] Caron J, Corbière M, Mercier C, Diaz P, Ricard N, Lesage A (2003). The construct validity of the client questionnaire of the Wisconsin quality of life index–a cross-validation study. Int J Methods Psychiatr Res.

[27] Hojjati-Abed E, Karbalaaei-nouri A, Rafiei H, Karimlou M (2010). The efficacy of psychosocial occupational therapy services on quality of life of chronic Pschiatric patents. Arch Rehabil..

[28] Matuszek S (2010). Animal-facilitated therapy in various patient populations: systematic literature review. Holist Nurs Pract.

[29] Berget B, Ekeberg Ø, Pedersen I, Braastad BO (2011). Animal-assisted therapy with farm animals for persons with psychiatric disorders: effects on anxiety and depression, a randomized controlled trial. Occup Ther Ment Health.

[30] Ambrosi C, Zaiontz C, Peragine G, Sarchi S, Bona F (2019). Randomized controlled study on the effectiveness of animal-assisted therapy on depression, anxiety, and illness perception in institutionalized elderly. Psychogeriatrics.

[31] Banks MR, Willoughby LM, Banks WA (2008). Animal-assisted therapy and loneliness in nursing homes: use of robotic versus living dogs. J Am Med Dir Assoc.

[32] Calvert MM (1989). Human-pet interaction and loneliness: a test of concepts from Roy's adaptation model. Nurs Sci Q.

[33] Bowlby J (1975). Attachment theory, separation anxiety, and mourning. Am Handb Psychiatry.

[34] Bernstein PL, Friedmann E, Malaspina A (2000). Animal-assisted therapy enhances resident social interaction and initiation in long-term care facilities. Anthrozoös.

[35] Barker SB, Knisely JS, McCain NL, Best AM (2005). Measuring stress and immune response in healthcare professionals following interaction with a therapy dog: a pilot study. Psychol Rep.

[36] Stefanini MC, Martino A, Bacci B, Tani F (2016). The effect of animal-assisted therapy on emotional and behavioral symptoms in children and adolescents hospitalized for acute mental disorders. Eur J Integr Med.

[37] Wood E, Ohlsen S, Thompson J, Hulin J, Knowles L (2018). The feasibility of brief dog-assisted therapy on university students stress levels: the PAwS study. J Ment Health.

[38] Coakley AB, Mahoney EK (2009). Creating a therapeutic and healing environment with a pet therapy program. Complement Ther Clin Pract.

[39] Crossman MK, Kazdin AE. Animal visitation programs in colleges and universities: An efficient model for reducing student stress. In: Handbook on animal-assisted therapy: Elsevier; 2015. p. 333–7.

[40] Antonioli C, Reveley MA (2005). Randomised controlled trial of animal facilitated therapy with dolphins in the treatment of depression. BMJ.

[41] Stasi M, Amati D, Costa C, Resta D, Senepa G, Scarafioiti C, Aimonino N, Molaschi M (2004). Pet-therapy: a trial for institutionalized frail elderly patients. Arch Gerontol Geriatr Suppl.

[42] Nordgren L, Engström G (2014). Animal-assisted intervention in dementia: effects on quality of life. Clin Nurs Res.

[43] Berget B, Braastad BO (2011). Animal-assisted therapy with farm animals for persons with psychiatric disorders. Ann Ist Super Sanita.

[44] Pedersen I, Martinsen EW, Berget B, Braastad BO (2012). Farm animal-assisted intervention for people with clinical depression: a randomized controlled trial. Anthrozoös.

